# Study of Hepatitis C Virus Detection Assays

**DOI:** 10.1016/j.amsu.2018.10.002

**Published:** 2018-10-06

**Authors:** Mithaq Sabeeh Khudur Al-Nassary, Batool Mutar Mahdi

**Affiliations:** a Al-Kindy Teaching Hospital, Laboratory Department, Iraq; b Al-Kindy College of Medicine, Head of HLA Research Unit, University of Baghdad, Iraq

**Keywords:** Hepatitis, Test, Screen, (Ab), Antibody, (Ag), Antigen, (HCV), Hepatitis C virus

## Abstract

**Background:**

Hepatitis C virus is a small, enveloped, positive-sense single-stranded RNA virus that causes and liver cancer like hepatocellular carcinoma and lymphomas.

**Aim of the study**: to assess different methods in diagnosis HCV infection.

**Patients and methods:**

A retrospective study of 426 patients was admitted to Al-Kindy Teaching Hospital, Baghdad-Iraq for surgical operations or renal dialysis from January-2015 to December-2016. Their serum tested for HCV Abs by rapid immunochromatography, Enzyme Linked ImunoSorbent Assay (ELISA), and RIBA test.

**Results:**

The study sample was 426 patients, their age was ranged from 15 to 65 years. Males were represented 58% and the rest were females. The serum of all samples has tested by rapid Immunochromatography test. Fifty percent of them showed positive results by this test and the rest were negative. Those fifty serum samples who were positive by Immunochromatography test were reexamined by ELISA test and showed 39out of 50 (78%) were true positive by ELISA test and the rest were negative (P = 0.0001).

The positive samples by ELISA have tested by RIBA test that showed 200(80%)were true positive in males and 130(74%)were true positive in females and the rest were false positive (P = 0.0001).

**Conclusions:**

Early screening of the high risk group of population by highly sensitive test is important to treat infected patients and prevent dissemination among population.

## Highlights

• Hepatitis C virus is a small, enveloped, positive-sense single-stranded RNA virus.
• To screen methods of different diagnostic tests against HCV.
• A retrospective study of 426 patients was admitted to Al-Kindy Teaching Hospital, Baghdad-Iraq for surgical operations or renal dialysis from January-2015 to December-2016. Their serum tested for HCV Abs by rapid immunochromatography, Enzyme Linked ImunoSorbent Assay (ELISA), and RIBA test.
• Fifty percent of them showed positive results by this test and the rest were negative. Then they have tested by ELISA test. 78% of patients who were positive by rapid test demonstrated true positivity by ELISA positive samples by ELISA have tested by RIBA test that showed 80% were true positive in males and 74% were true positive in females.
• Early screening of the population by highly sensitive test is important.

## 1 Introduction

Hepatitis C virus (HCV) is a small, enveloped, positive-sense single-stranded RNA virus belongs to *Flaviviridae* family. It is the cause of hepatitis C infection and liver cancer like hepatocellular carcinoma (HCC) and lymphomas [1]. It is classified into six genotypes [[1], [2], [3], [4], [5], [6]] according to genetic differences in the nucleotide locations over the complete genome and subtypes 1a and 1b are more common worldwide and represent 60% of all cases [2]. Hepatitis C virus is mainly a blood-borne virus and the common risk group was injecting drug users, recipients of blood and its products, patients on haemodialysis, nosocomial transmission by inadequate sterilization and cultural or ritual practices like ritual scarification, tattooing and acupuncture [3]. There is a proof that suggests exosomes can transfer genetic materials of HCV between cells [4]. It is very important to screen and diagnose HCV infection in those high risk groups and can treat them early with antiviral therapy. Infected people with HCV are at a high risk of chronic liver disease, cirrhosis and hepatocellular carcinoma [5]. So screening of HCV infection is important for early treatment of disease with antiviral drugs which is more effective than later in the course of the disease and reduces the rate of viral transmission [6]. The American Association for the Study of Liver Diseases (AASLD) advises screening for high risk group of population [7]. Diagnosis of HCV can be done by indirect serologic tests that detect antibodies against HCV and direct test that detect, quantify and components of viral RNA particles [8]. Core antigen of HCV used as a screening method for HCV viremia in individuals living in endemic area and a marker to assess active HCV infection, It is a good surrogate marker for HCV RNA [9]. All health care workers need to understand how to establish or exclude a diagnosis of HCV infection and to interpret the tests correctly. This study aims to discuss the screening methods of diagnostic tests and interpretation of available assays.

### 1.1 Patients and methods

A retrospective study of 426 patients was admitted to Al-Kindy Teaching Hospital, Baghdad-Iraq for surgical operations or renal dialysis from January-2015 to December-2016. Their serum tested for HCV Abs by rapid immunochromatography, Enzyme Linked ImunoSorbent Assay (ELISA), and RIBA test.

The revision was accepted by the Al-Kindy College of Medicine and Al-Kindy Teaching Hospital. Data was collected from them including demographic information age, sex, marital status, occupation, residential status.

### 1.2 Serological testing

A total of 426 serum samples were tested for HCV Abs using Rapid Immunochromatography test (CTK/USA) by loading the serum sample and migrate through nitrocellulose membrane forming a colored line for a positive test.

Then examined by enzyme-labeled immune sorbent test (ELISA) for detection anti HCV— Abs (IgG) (Foresight-EIA-USA). The principle of this test, the microwell is coated with Ags then the serum will be added that contains Abs leads to formation a complex. After incubation, washing was done and enzyme conjugated with Abs were added. After incubation and washing were done; substrate A and B were added. The color was formed and the reaction was stopped by sulfuric acid. The results were interpreted after reading with microplate reader at 450 nm within 30 min. Samples with optical densities below the cutoff were recorded as negative, those with optical densities between 10% below and 10% above the cutoff were equivocal, and all others were positive. If the sample's absorbance was within 10% of the cutoff level, the sample was retested and classified according to the retest result.

Lastly the test was confirmed by Recombinant Immunoboltting Assay (RIBA) test (Chirsun/USA) for detection HCV Abs. This test utilizes four recombinant HCV Ags (c100p, c33c, p22p and NS-5) that immobilized on the test strip and Abs in the patients serum will bind to it giving either non-reactive, intermediate or reactive).

### 1.3 Statistical analysis

Data were analyzed statistically using:

- Descriptive statistics: frequencies, mean and standard deviation.
- Inferential statistics: Chi-square tests and fisher exact test.

All of these were done using MiniTab statistical software program 13.20. A P— value ≤ 0.05 was considered to be significant.

### 1.4 Strocss

The work has been reported in line with STROCSS criteria and site the paper as follows:

Al-Nassary MSK and Mahdi BM. Study of Hepatitis C Virus Detection Assays.

## 2 Results

The study sample was 426 patients, their age was ranged from 15 to 65 years (35 ± 2.1) (X±SEM). All of them were from Baghdad province. Males were 250 (58%) and the rest were females 176 (42%) (0.0001)as shown in Table 1.

**Table 1:** Demographic data of the patients with HCV.

Total Sample patients No = 426	Male		Female		P— value
	No	%	No	%	
Sex	250	58	176	42	0.0001
Age (years) (X±SEM)	35 ± 1.1		34 ± 1.5		N.S.
Marital status married	240(96%)		171(97.15%)		N.S.
Governmental Occupation	150(60%)		100(56.81%)		N.S.
Residential status in urban	220(88%)		130(73.86%)		N.S.

The serum of all samples has tested by rapid Immunochromatography test. Fifty percent of the showed positive results by this test and the rest were negative. Then they have tested by ELISA test. Out of fifty 39(78%) of patients who were positive by rapid test demonstrated true positivity by ELISA and the rest were false positive by rapid test which is significant (P = 0.0001)(Table 2).

**Table 2:** Positive sample detection by Rapid-Test and confirming by ELISA.

Positive sample by Rapid test*		ELISA Positive**		False Positive***		P-value
No	%	No	%	No	%	
50	(100%)	39	(78%)	11	(22%)	0.0001

*Rapid test = immunochromatographic assay.

**The samples which positive by Rapid test and ELISA.

***The samples which positive by Rapid test but negative by ELISA.

The negative serum for HCV Abs (50)(100%) that tested by rapid test have tested by ELISA and showed 49(98%)of them were true negative and only one sample was false negative (2%) as mentioned in Table 3.

**Table 3:** Negative sample detection by Rapid-test and confirming by ELISA.

Negative sample by Rapid test		ELISA Negative*		False Negative**		P-value
No	%	No	%	No	%	
50	(100%)	49	(98%)	1	(2%)	0.0001

*The samples which negative by Rapid test and ELISA.

**The samples which negative by Rapid test but positive by ELISA.

The positive samples by ELISA have tested by RIBA test that showed 200(80%)were true positive in males and 130(74%) were true positive in females and the rest were false positive (P = 0.0001) as shown in Table 4–a and b.

**Table 4:** The positive sample detected by ELISA and confirmed by RIBA in males (a) and females (b).

a						
Male						P-value
ELISA		RIBA*		False positive**		
No	%	No	%	No	%	
250	(100%)	200	(80%)	50	(20%)	0.0001
b						
Female						P-value
ELISA		RIBA*		False positive**		
No	%	No	%	No	%	
176	(100%)	130	(74%)	46	(26%)	0.0001

*RIBA = Recombinant Immunoboltting Assay.

**The samples were positive by ELISA but negative by RIBA.

## 3 Discussion

Infection of liver with hepatitis C virus can lead to acute or chronic hepatitis, liver cirrhosis, end-stage liver disease, and lastly hepatocellular carcinoma [10]. Study of Gower E et al., 2014 [11] was done in Center of disease analysis in Louisville, USA reported anti HCV prevalence in eighty seven countries and the total global viraemic HCV infections were estimated at 80 (64–103) million infections. These Abs found either due to active infection or resolved infection after treatment with drugs [12]. The diagnosis of HCV can be done by indirect serologic tests detecting specific antibody to HCV and direct tests that quantify HCV viral particles like HCV RNA and core antigen. In this study we tested and screened the patients who had prepared to surgical operations, renal dialysis and gastroscopy for detection anti HCV Abs using three methods. The advantages of this are to limit the spread of the virus among individuals and early treatment of infected person [13,14]. We found that rapid immunochromatography test had false positive (22%) and false negative (2%). The second test was ELISA that decreases the window phase to 17 days [15]. ELISA test showed false positive 20% in males and 26% in females. These percentages were depending on the manufacturer that synthesis the kits and from which generation of ELISA used because there was four generations of ELISA depending on viral Ags used. The first generation of ELISA used since 1989 which incorporated the recombinant c100-3 epitope from the NS4 region. In 1992, second generation of ELISA appeared which used epitopes c22-3 and c33c from the HCV core and NS3 regions, respectively. The third generation used reconfigured core and NS3 antigens and in addition a newly incorporated antigen from the NS5 region. The last fourth generation of ELISA is those that detect HCV capsid antigen as well as antibodies to the core, NS3, NS4, and NS5 regions of the virus(15). The sensitive test was RIBA used in as supplemental test to confirm serological reactivity by ELISA. It showed 80% true positive in males and 74% in females and the percentages of false positive were decreased. Studies have showed that higher the anti-HCV antibody titer in patient's serum; more likely to be true positive than false positive. It acts as indirect marker for confirmation of serological results [16].

Other study demonstrated that molecular testing of HCV RNA is a confirming test for HCV infection and monitor treatment but this test needs skilled and trained laboratory staff, sophisticated equipment and devices, and expensive reagents [17]. So testing of HCV core antigen is useful for the management of patients with chronic hepatitis C virus [18] and evaluate response to treatment [19,20]. Therefore, a simple, cost-effective rapid immunochromatographic test is feasible for routine HCV screening would be ideal for developing countries like Iraq. This primary test for diagnosis of HCV infection, other confirmatory tests is still needed to prove the diagnosis. The results of this test suggest that antibodies against HCV Ags had a poor correlation with HCV RNA and are appropriate for primary screening and needs repeat for positive samples by ELISA and RIBA due to their high sensitivity. RIBA can detect Abs against HCV Ags(c100p,c33c, p22p and NS-5). Wasitthankasem R., et al., 2017 [21] Showed that detection of HCV Ag as a reliable marker for diagnosis of active HCV infection with a good correlation with the viral RNA irrespective of genotypes. It also acts as a marker for detection HCV infection and reduces the sample number requiring further confirmatory RNA assays. Thus, decrease the cost and financial burden for screening of HCV. The goal of World Health Organization (WHO) by the year 2030 is eradication of viral hepatitis by early diagnosis and treatment by inexpensive drugs [22,23]. It is important to know the interpretation of the result by health laboratory workers in order to give a true diagnosis of the disease and prevent any insult to hepatic tissues because of absence of vaccine. The differences of our results with other study may be due to sample size used in the study and the type of kit and manufacturing company that synthesized the kit which differ in its sensitivity and specificity. Thus, it's preferable to do this study on large number size of the patients.

## 4 Conclusions

Early screening of the high risk group of population by highly sensitive test is important to treat infected patients and prevent dissemination among population.

## Provenance and peer review

Not commissioned, externally peer reviewed.

## Consent

Yes.

## Ethical approval

Yes.

## Source of funding

None.

## Author contribution

Dr. Batool wrote paper.

Dr. Methaq collect and did the tests.

## Conflicts of interest

Non.

## Registration of research studies

UIN = 4078.

## Guarantor

Consultant Dr Ali Ghalib Mutar Mahdi.
